# Management insulin dosing for diabetes using a partially observable Markov decision process with missing data imputation

**DOI:** 10.1038/s41598-026-58337-w

**Published:** 2026-06-18

**Authors:** Jiao Xiang, Haiyan Yu, Li Luo, Shanshan Liu

**Affiliations:** 1 Business School, Sichuan University, 1st Ring Road, Sichuan Chengdu, 610065 China; 2 Department of Psychiatry & Key Laboratory of Major Brain Disease and Aging Research (Ministry of Education) & Psychiatric Center, The First Affiliated Hospital of Chongqing Medical University, No. 1 Youyi Road, Yuanjiagang, Yuzhong District, Chongqing, 400016 China; 3 Institute of Hospital Management, West China Hospital of Sichuan University, Sichuan University, No. 17 Renmin South Road, Sichuan Chengdu, 610041 China

**Keywords:** Partially observable Markov decision process, Bridge-based adjusted Metropolis-Hastings algorithm, Missing data imputation, Continuous glucose monitoring, Policy disagreement, Computational biology and bioinformatics, Diseases, Health care, Mathematics and computing, Medical research

## Abstract

Missing data in continuous glucose monitoring (CGM) poses a significant challenge for applying sequential decision-making models to diabetes management. This study evaluates how missing-data imputation affects downstream Partially Observable Markov Decision Process (POMDP)-based policy outputs using real CGM trajectories from the Stanford Continuous Glucose Monitoring Database. Three imputation methods are compared: mean imputation, linear interpolation, and a bridge-based adjusted Metropolis-Hastings (M-H) algorithm. The adjusted M-H algorithm incorporates a local temporal bridge, Markovian state-transition information, and a smoothness constraint to generate model-compatible imputations. Numerical experiments are conducted under two missingness scenarios, random missingness and block missingness, with missing rates of 5%, 15%, and 25%. The methods are evaluated using mean squared imputation error (MSIE), policy disagreement rate, and absolute reward gap relative to the complete-data POMDP benchmark. The results show that mean imputation produces substantially larger reconstruction errors and greater downstream POMDP deviations across missingness scenarios. Linear interpolation and adjusted M-H both preserve CGM trajectories and POMDP-derived policy outputs much better than mean imputation. Linear interpolation achieves slightly lower global MSIE under random missingness, whereas adjusted M-H shows comparable POMDP-level performance and local advantages in nonlinear postprandial trajectories and block-missing segments. These findings suggest that temporally informed imputation methods are preferable to mean imputation for incomplete CGM data, and that adjusted M-H provides a model-compatible alternative for preserving sequential decision outputs under partially observed glucose trajectories.

## Introduction

Diabetes remains one of the most common chronic diseases, with about 589 million people living with it in 2021, and the global burden will increase to 853 million people in 2050^1^. For most patients with diabetes, lifelong insulin therapy is required to stabilize and control blood glucose levels^2^. However, the dynamic nature of diabetes management necessitates sequential decision-making, where each treatment adjustment depends on the patient’s current physiological state and prior responses.

The Partially Observable Markov Decision Process (POMDP) provides a powerful mathematical framework for modeling sequential decision-making under uncertainty^3,4^, making it particularly suitable for diabetes management. By maintaining a belief state, a probability distribution over the patient’s unobserved true health status, POMDP models can integrate historical observations to generate sequential policy outputs that maximize model-defined rewards under a specified decision structure. Currently, this method is also increasingly being applied in chronic disease management^5–9^ and medical treatment decision-making. A Markov Decision Process (MDP) assumes that the decision maker can fully observe the true state of the system. However, in many healthcare settings, including diabetes management, the exact physiological state is not directly observable. POMDP generalizes MDP by allowing the agent to maintain a belief distribution over states based on partial observations and historical actions^22^. This makes POMDP particularly suitable for chronic disease management where measurements are noisy or missing.

A critical prerequisite for applying POMDP-based sequential decision models to simulation related to continuous glucose monitoring (CGM) or decision-support research is the availability of continuous observational data to enable reliable belief updating and policy execution. This necessitates regular monitoring of blood glucose levels. In the context of diabetes management, CGM is widely recognized as a supplementary tool, which can offer detailed insights into blood glucose fluctuations as depicted in the internationally standardized dynamic blood glucose curve compared to traditional care. This allows both users and clinicians to make more informed decisions, potentially improving blood glucose outcomes and the quality of life for individuals with diabetes^10^.

In clinical trials, the effectiveness of intervention measures in improving blood glucose levels is evaluated using two methods-over the entire intervention period (3-6 months) and over a shorter time range (2-3 weeks). However, both methods often encounter issues with incomplete or missing datasets^11^. This is because CGM can be prone to errors and inconsistent usage in real-world settings, leading to connection losses or sensor failures. Users may also lose data when they remove their devices during periods of intense activity or for other reasons, contributing to approximately 10% of missing CGM data^12^. This data incompleteness poses a significant challenge: standard POMDP frameworks assume a fixed observation structure and are not inherently designed to handle irregular or missing data. Therefore, how to effectively perform sequential decision-making using POMDP in the presence of missing CGM data becomes a critical issue that must be addressed to assess the robustness of model-based sequential decision-making under incomplete CGM observations. Simple methods such as mean imputation^19^ ignore the temporal structure of glucose dynamics, whereas linear interpolation^25^ uses neighboring observations and is often effective for short and smooth missing gaps. However, linear interpolation may be less suitable when missing values occur during nonlinear postprandial excursions, delayed glucose peaks, or rapid transitions between glucose states. These settings motivate the evaluation of model-compatible imputation methods that can incorporate both local temporal information and Markovian state-transition assumptions.

The goal of this study is to evaluate how missing-data imputation affects downstream POMDP policy outputs using real CGM trajectories. Specifically, we compare mean imputation, linear interpolation, and a bridge-based adjusted M-H algorithm under both random and block missingness scenarios at missing rates of 5%, 15%, and 25%. The proposed adjusted M-H method combines a local temporal bridge, Markovian state-transition information, and a smoothness constraint to generate model-compatible imputations. We evaluate the methods using three evaluation metrics. This design allows us to examine whether imputation methods that preserve local glucose dynamics can better maintain POMDP-derived policy outputs when CGM observations are incomplete.

## Method

### Data source

The dataset employed in this study is derived from the Stanford Continuous Glucose Monitoring Database^26,27^, a publicly available dataset containing CGM measurements collected during standardized food challenge experiments. The CGM data file contains 23,520 glucose measurements from 38 subjects. Each observation records the glucose value, subject identifier, food condition, mitigator information, replicate number, and time in minutes since the start of the experiment. The original glucose values are reported in mg/dL and were converted to mmol/L by dividing by 18 to align with the glucose-state definitions used in the POMDP framework.

Complete CGM trajectories are generated by grouping observations by subject, food condition, food type, and replicate number. This preprocessing step resulted in 588 complete CGM sequences, with each sequence containing 40 measurements from 25 to 170 min relative to the start of the food challenge at 5-minute intervals. These complete sequences served as the reference data for the missing-data experiments.

Specifically, we set different missing patterns of CGM observations at missing rates of 5%, 15%, and 25%. Two missing-data scenarios are considered. In the random missingness scenario, missing values are generated by randomly removing CGM observations at the target missing rates. Because random deletion may occasionally generate short consecutive missing segments but does not control their length or frequency, we additionally considered a block missingness scenario. In this scenario, missing values are generated as consecutive blocks of 2-6 observations while maintaining the same target missing rates of 5%, 15%, and 25%.

### Adjusted M-H algorithm

The adjusted M-H algorithm is used as a model-compatible imputation method for missing CGM observations. Unlike simple mean imputation, which ignores temporal dependence, the adjusted M-H algorithm incorporates local temporal structure and Markovian state-transition information. For each consecutive missing segment, a local temporal bridge is first constructed using the neighboring observed CGM values. The M-H sampler is then used to generate imputed values around this local bridge while incorporating a Markovian transition prior and a smoothness constraint.

Let $\:y=({y}_{1},\dots\:,{y}_{T})$denote a CGM trajectory and $\:\mathcal{M}$ the set of artificially missing positions. For a missing segment bounded by observed values, a local bridge trajectory $\:{\stackrel{\sim}{y}}_{t}$ is constructed. In the simplest case, $\:{\stackrel{\sim}{y}}_{t}$ corresponds to the linear interpolation value between the two neighboring observed CGM values. To allow mild local curvature, a local quadratic correction is also considered, and the bridge center is expressed as.

$$\:{\stackrel{\sim}{y}}_{t}={y}_{t}^{L}+\omega\:\cdot\:\mathrm{c}\mathrm{l}\mathrm{i}\mathrm{p}\left({y}_{t}^{Q}-{y}_{t}^{L},-c,c\right),t\in\:\mathcal{M},$$

where $\:{y}_{t}^{L}$ is the linear bridge value, $\:{y}_{t}^{Q}$ is the local quadratic fitted value, $\:\omega\:$ is the curvature weight, and * c* controls the maximum allowed local adjustment. This construction allows the adjusted M-H algorithm to remain close to the local observed CGM pattern while permitting limited nonlinear correction.

The target distribution of the missing values is defined by three components: a local temporal bridge likelihood, a Markovian state-transition prior, and a smoothness penalty. Specifically, the log target function for a missing segment is written as

$$\:\log \pi \:\left( {y_{{\mathcal{M}}} } \right) = - \frac{1}{{2\sigma \:_{b}^{2} }}\sum {\:_{{t \in \:{\mathcal{M}}}} } (y_{t} - \mathop y\limits^{\sim }_{t} )^{2} + \beta \:\sum {\:_{t} } \log \left[ {q(s_{t} ,s_{{t + 1}} ) + \in } \right] - \lambda \:\sum {\:_{t} } (\Delta \:^{2} y_{t} )^{2} ,$$

where $\:{\stackrel{\sim}{y}}_{t}$ is the local bridge value, $\:q({s}_{t},{s}_{t+1})$is the Markov transition probability from glucose state $\:{s}_{t}$ to $\:{s}_{t+1}$, $\:\beta\:$ controls the contribution of the Markov transition prior, and $\:\lambda\:$ controls the smoothness penalty. The final term penalizes abrupt second-order fluctuations in the local imputed trajectory. A small constant *∈* is added to avoid taking the logarithm of zero.

For each missing segment, candidate values are proposed jointly using a Gaussian random-walk proposal,

$$\:{y}_{\mathcal{M}}^{*}={y}_{\mathcal{M}}+\eta\:,\eta\:\sim\:N(0,{\sigma\:}_{p}^{2}I),$$

where $\:{\sigma\:}_{p}$ is the proposal standard deviation. Because the proposal distribution was symmetric, the M-H acceptance probability was calculated as

$$\:\alpha\:=\mathrm{m}\mathrm{i}\mathrm{n}\left\{1,\mathrm{e}\mathrm{x}\mathrm{p}\left[\mathrm{l}\mathrm{o}\mathrm{g}\pi\:\left({y}_{\mathcal{M}}^{*}\right)-\mathrm{l}\mathrm{o}\mathrm{g}\pi\:\left({y}_{\mathcal{M}}\right)\right]\right\}$$

The proposed missing segment is accepted with probability $\:\alpha\:$; otherwise, the current segment values are retained. After burn-in, the retained M-H samples are averaged to obtain the final imputed values. In the experiments, the same M-H parameters are used across missing-rate scenarios to ensure comparability.

It should be noted that although the local bridge uses linear interpolation as a temporal center, the adjusted M-H algorithm is not identical to deterministic linear interpolation. The final imputed values are obtained through M-H sampling under a target function that also incorporates Markovian glucose-state transitions and local smoothness constraints. Therefore, the method provides a model-compatible imputation approach within the POMDP framework.

In addition, two comparator methods are included. Mean imputation replaces each missing CGM value with the mean of the observed glucose values from the corresponding complete trajectory. Linear interpolation estimates each missing value using the two neighboring observed values surrounding the missing segment. For a missing segment of length * m* bounded by observed values $\:{y}_{i}$ and $\:{y}_{i+m+1}$, the * k*-th missing value is imputed as

$$\:{\widehat{y}}_{i+k}={y}_{i}+\frac{k}{m+1}\left({y}_{i+m+1}-{y}_{i}\right),k=1,\dots\:,m.$$.

Linear interpolation is applied to internally bounded missing segments and serves as a strong temporal baseline for short CGM gaps.

### Scenario-based POMDP model with missing data

To evaluate how missing CGM data and different imputation methods influence downstream sequential decision-making, we develop an infinite-horizon discounted scenario-based POMDP model to evaluate model-based insulin-intensity policy outputs using imputed CGM observations, where the transition probabilities, observation probabilities, and reward function are pre-specified as fixed scenario parameters rather than estimated from the limited simulated data or from observed clinical action-response records. Firstly, considering a homogeneous Markov chain $\:{\left({y}_{t}\right)}_{t\in\:\left[0,\:+\infty\:\right]}$ with $\:t\:$ discrete time points. The POMDP model consists of seven tuples: $\:\left[S,\:A,\:P,\:R,\:Y,\:K,\:\gamma\:\right]$, where * S* denotes the latent glucose-related health states, * A* is a set of actions representing vary insulin intensities, $\:P:S\times\:A\times\:S\to\:\left[\mathrm{0,1}\right]$ is state transition probability matrices, * R* denotes the reward function, * Y* is a set of observations, $\:K:S\times\:A\times\:Y\to\:\left[\mathrm{0,1}\right]$ is observable probability matrix, and $\:\gamma\:\in\:\left[\mathrm{0,1}\right]$ is a discount factor representing the influence of the current state on the successive states gradually decreases.

The *state space* *S* is defined by four discretized glucose-related categories based on blood glucose values: $\:{s}_{1}=\left(\mathrm{0,7.8}\right]mmol/L$, $\:{s}_{2}=\left(\mathrm{7.8,11.1}\right]mmol/L$, $\:{s}_{3}=\left(\mathrm{11.1,14.0}\right]mmol/L$ and $\:{s}_{4}=\left(\mathrm{14.0,30.0}\right]mmol/L$. The categories are used to represent the latent glucose-related health state in the numerical study.

The *observation space* *Y* uses the same four glucose intervals: $\:{y}_{1}=\left(\mathrm{0,7.8}\right]mmol/L$, $\:{y}_{2}=\left(\mathrm{7.8,11.1}\right]mmol/L$, $\:{y}_{3}=\left(\mathrm{11.1,14.0}\right]mmol/L$ and $\:{y}_{4}=\left(\mathrm{14.0,30.0}\right]mmol/L$. For each CGM value, the corresponding observation category is determined according to the interval into which the glucose value falls. In the presence of missing CGM values, the missing observation is first imputed using either the adjusted M-H algorithm or the comparator imputation method. The completed glucose trajectory is then discretized into the four observation categories defined above and used as the observation input for the POMDP model. In this way, different imputation methods generated different completed observation sequences, while the underlying POMDP parameterization is fixed across methods.

The *action space* *A* represents four simulator-defined insulin-intensity categories: $\:A=\{{a}_{1},{a}_{2},{a}_{3},{a}_{4}\}$, where $\:{a}_{1}$ denotes no insulin intensity, $\:{a}_{2}$ denotes low insulin intensity, $\:{a}_{3}$ denotes moderate insulin intensity and $\:{a}_{4}$ denotes high insulin intensity. These actions should be interpreted as model-based insulin-intensity categories in the numerical experiment rather than as real-world clinical insulin-dose prescriptions. No specific clinical insulin units are assigned to these action categories.

The *transition probability* $\:P\left(s{\prime\:}|s,a\right)$ represents the probability of moving from current sates * s* to next state $\:s{\prime\:}$ after taking action * a*. Since the available data did not contain observed action-specific treatment-response records, these transition probabilities are pre-specified rather than estimated from the glucose trajectories. The full transition probability matrices used in the analysis are provided in Table 1.

**Table 1:** Transition probability matrices.

	$\:{a}_{1}$				$\:{a}_{2}$				$\:{a}_{3}$				$\:{a}_{4}$			
	$\:{s}_{1}$	$\:{s}_{2}$	$\:{s}_{3}$	$\:{s}_{4}$	$\:{s}_{1}$	$\:{s}_{2}$	$\:{s}_{3}$	$\:{s}_{4}$	$\:{s}_{1}$	$\:{s}_{2}$	$\:{s}_{3}$	$\:{s}_{4}$	$\:{s}_{1}$	$\:{s}_{2}$	$\:{s}_{3}$	$\:{s}_{4}$
$\:{s}_{1}$	0.4	0.6	0	0	0.5	0.5	0	0	0.6	0.4	0	0	0.7	0.3	0	0
$\:{s}_{2}$	0.2	0.4	0.4	0	0.3	0.4	0.3	0	0.4	0.4	0.2	0	0.6	0.3	0.1	0
$\:{s}_{3}$	0	0.2	0.4	0.4	0	0.3	0.4	0.3	0	0.4	0.4	0.2	0	0.6	0.3	0.1
$\:{s}_{4}$	0	0	0.6	0.4	0	0	0.7	0.3	0	0	0.8	0.2	0	0	0.9	0.1

The *observation probability* $\:K\left(y|s,a\right)$ describes the probability of observing category * y* when the underlying glucose-related is * s*. In this study, the observation probability matrix is also pre-specified as part of the numerical POMPD setting. The full observation probability matrix is provided in Table 2.

**Table 2:** Observation probability matrices.

	$\:{a}_{1}$				$\:{a}_{2}$				$\:{a}_{3}$				$\:{a}_{4}$			
	$\:{s}_{1}$	$\:{s}_{2}$	$\:{s}_{3}$	$\:{s}_{4}$	$\:{s}_{1}$	$\:{s}_{2}$	$\:{s}_{3}$	$\:{s}_{4}$	$\:{s}_{1}$	$\:{s}_{2}$	$\:{s}_{3}$	$\:{s}_{4}$	$\:{s}_{1}$	$\:{s}_{2}$	$\:{s}_{3}$	$\:{s}_{4}$
$\:{s}_{1}$	0.5	0.5	0	0	0.6	0.4	0	0	0.7	0.3	0	0	0.8	0.2	0	0
$\:{s}_{2}$	0.3	0.4	0.3	0	0.4	0.4	0.2	0	0.5	0.4	0.1	0	0.6	0.3	0.1	0
$\:{s}_{3}$	0	0.3	0.4	0.3	0	0.4	0.4	0.2	0	0.5	0.3	0.2	0	0.6	0.3	0.1
$\:{s}_{4}$	0	0	0.5	0.5	0	0	0.6	0.4	0	0	0.7	0.3	0	0	0.8	0.2

The *immediate reward* *R(s,a)* is defined as a pre-specified state-action reward matrix. Specifically, higher rewards are assigned to more favorable glucose-related states, while lower rewards are assigned to less favorable states. Within each state, lower insulin-intensity actions are assigned relatively higher immediate rewards than higher insulin-intensity actions. The reward values should be interpreted as internal model-based quantities under the assumed POMDP parameterization rather than as validated clinical endpoints or direct evidence of clinical benefit. The full reward is provided in Table 3.

**Table 3:** The immediate reward with respect to state and action.

	$\:{a}_{1}$	$\:{a}_{2}$	$\:{a}_{3}$	$\:{a}_{4}$
$\:{s}_{1}$	10	7	4	1
$\:{s}_{2}$	7	10	7	4
$\:{s}_{3}$	4	7	10	7
$\:{s}_{4}$	1	4	7	10

The *discount factor* is set to $\:\gamma\:=0.9$, which determines the relative weight assigned to future rewards. The same discount factor is used for all imputation methods and missing-rate scenarios.

**Fig. 1:**
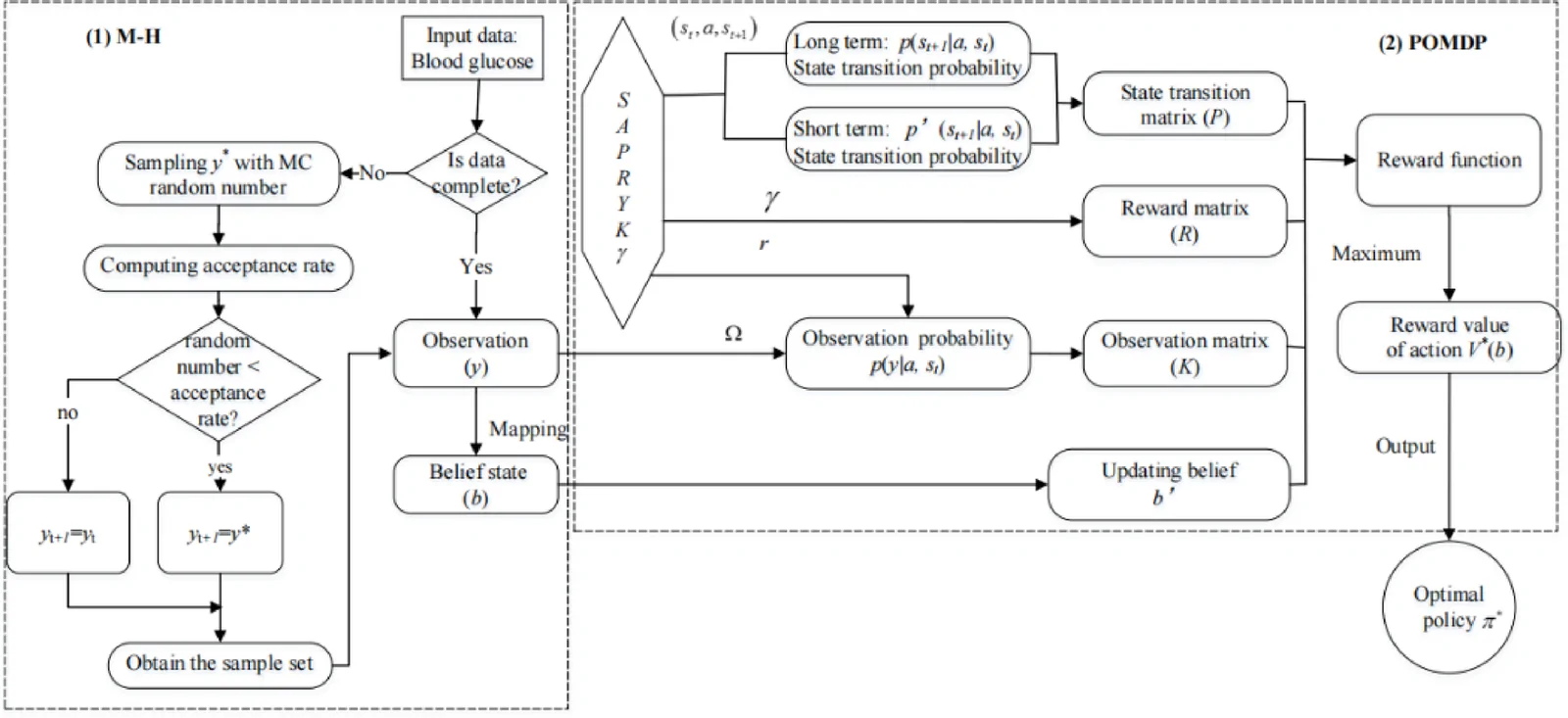
Flowchart of POMDP model based on imputed missing blood glucose data.

Figure 1 presents a flowchart delineating the process of a POMDP model designed to handle missing blood glucose data. The left segment of the figure depicts the adjusted M-H algorithm, while the right segment illustrates the POMDP model equipped to manage missing data. The process initiates with the input of data and an assessment of its completeness. In the event of complete data, it is directly incorporated into the model for resolution. Conversely, if the data is incomplete, the adjusted M-H algorithm is employed to supplement the missing data. Subsequently, the supplemented “complete” data is incorporated into the model on the right. Within the right segment, the parameters of each element in the model are input sequentially. The model is then resolved using dynamic programming principles, culminating in the acquisition of the optimal strategy.

In this numerical POMDP setting, the “belief state” summarizes the probability distribution over glucose-related states, while the “action” denotes a simulator-defined insulin-intensity category rather than a real-world insulin-dose prescription. In the realm of clinical practice, physicians typically monitor patients over a specified duration (spanning several hours) to assess their health status. However, in the process of state measurement and data collection, missing data $\:{y}_{t}^{mis}$ may occur undoubtedly. In this study, due to the assumption that data is missing at random, the missing data in the CGM dataset can be explained by the observation results in that dataset. In a recursive model, the attainment of the maximum value necessitates the “completeness” of the observation. To address the issue of missing values, this study enhances the M-H algorithm by integrating existing detailed equilibrium conditions.

**Fig. 2:**
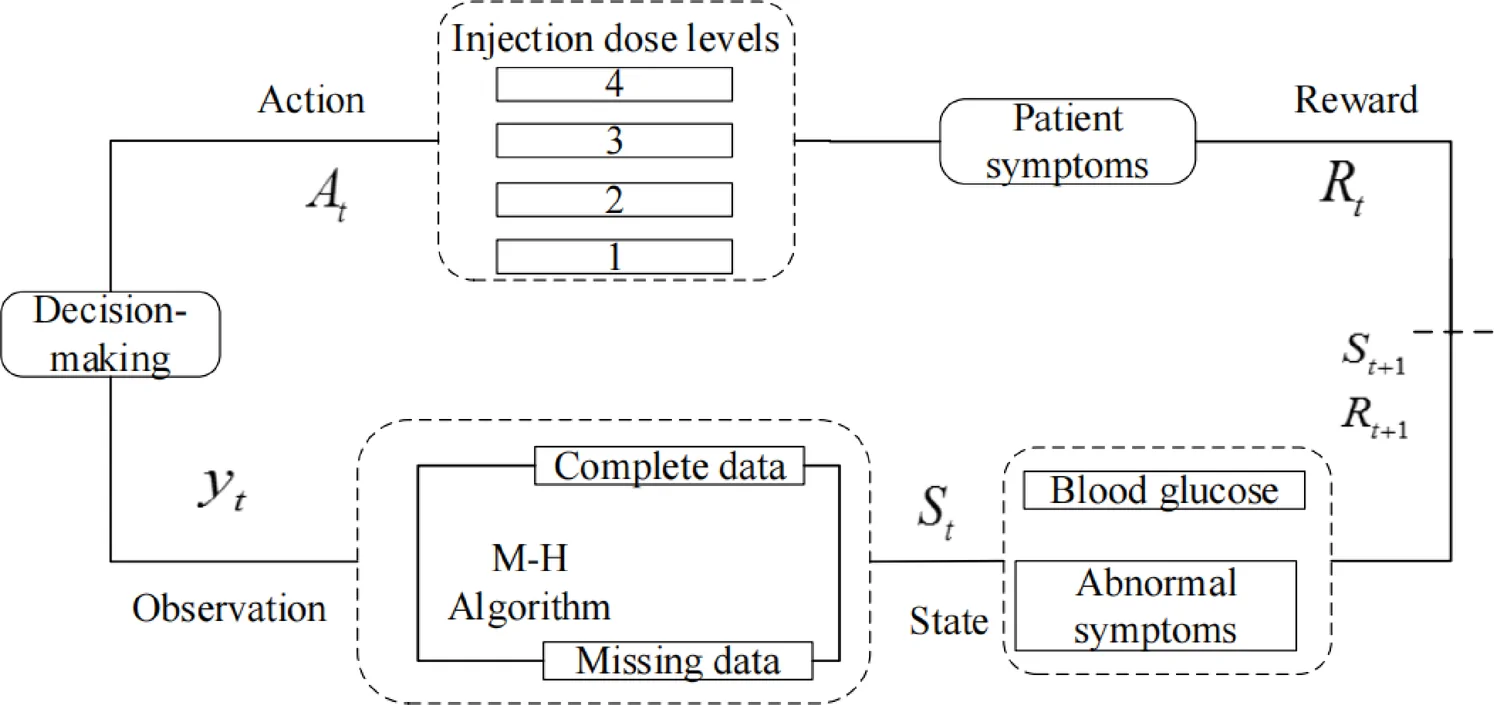
Application of POMDP model based on missing blood glucose data in the management of diabetes.

Figure 2 shows the process of diabetes management with POMDP model in the absence of data. Observation * y* represents the blood glucose level with missing data, which is divided into four intervals. For example, if the observed value of blood glucose is 12.88, then $\:{y}_{3}$ is used to represent the corresponding observation. However, observations are usually incomplete (e.g., 12.88 is missing).

## Results

### Visualization of POMDP policy

The POMDP is implemented using the R package “POMDP”^21^. For each missing-rate scenario and imputation method, the completed glucose data are mapped to observation categories and incorporated into the same pre-specified POMDP structure. The resulting policies and cumulative rewards are then compared across imputation methods. Because the POMDP parameters are pre-specified, the policy outputs should be interpreted as model-based results under the assumed parameterization rather than as clinically validated insulin recommendations. The initial belief state is set as $\:{b}_{0}=(0.2,\:0.5,\:0.2,\:0.1)$, representing a patient whose most likely initial condition is moderate hyperglycemia. During policy execution, the belief state is updated according to the POMDP belief-update rule after each action and observation.

**Fig. 3:**
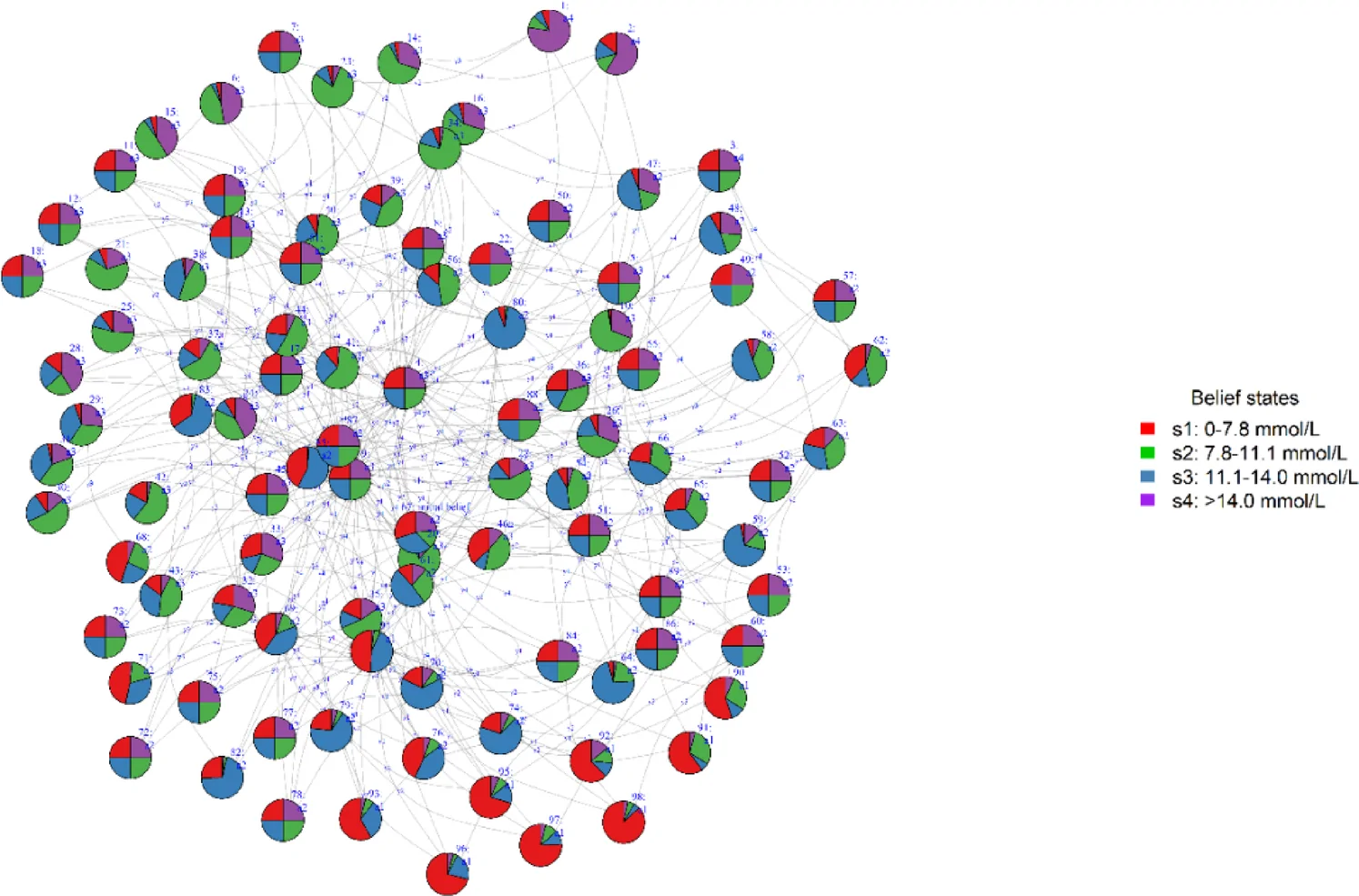
Policy graph of the scenario-based POMDP model. Each node represents a belief state, and the pie chart shows the probability distribution across four glucose states. Node labels indicate the optimal action, and edge labels indicate glucose observations. The policy graph visualizes how the POMDP maps uncertain glucose-related belief states to insulin-intensity actions.

Figure 3 shows the policy graph generated from the solved scenario-based POMDP model. The graph summarizes the mapping from belief states to optimal actions under the predefined transition, observation, and reward structures. Each node corresponds to a belief state, and the pie chart within each node represents the probability distribution across the four discretized glucose states. The action label associated with each node indicates the optimal insulin-intensity action selected by the POMDP policy, while the directed edges represent belief transitions after receiving possible glucose observations $\:{y}_{1}$-$\:{y}_{4}$.

For example, the initial belief state was set as $\:{b}_{0}=\left(\mathrm{0.20,0.50,0.20,0.10}\right)$, indicating that the patient was considered most likely to be in state $\:{s}_{2}$, while still retaining uncertainty across the other glucose states. Under this belief distribution, the POMDP selected the corresponding optimal action according to the expected discounted reward. After a new glucose observation is received, the belief state is updated through the transition and observation models, and the policy may move to another node with a different action. Therefore, the selected action is not determined solely by the most probable glucose state, but by the complete belief distribution and the expected future reward.

This policy graph illustrates how the POMDP model handles uncertainty in partially observed CGM information. Belief states with similar dominant glucose categories may still lead to different actions if their probability distributions over the remaining states differ. Conversely, different belief states may select the same action when their expected future rewards are similar. Thus, the graph provides a visual representation of the decision logic of the scenario-based POMDP model, rather than a deterministic rule based only on observed glucose values.

To avoid overinterpretation, the actions in this graph should be regarded as model-defined insulin-intensity categories under the predefined scenario parameters, not as clinically validated insulin dosing recommendations.

### Evaluation metrics for performance comparison under missing CGM data

To assess the robustness of the proposed framework under incomplete CGM observations, we compare mean imputation, linear interpolation, and the adjusted M-H algorithm on the Stanford CGM dataset. For each complete CGM sequence, artificial missing values are introduced at three missing rates: 5%, 15%, and 25%. Two missingness scenarios are considered. The first is random missingness, in which missing values are randomly generated across the CGM trajectory. The second is block missingness, in which consecutive missing segments are generated to represent short periods of continuous signal loss that may occur in real CGM monitoring.

In order to compare the imputation methods, three evaluation metrics are introduced. The first metric is Mean Squared Input Error (MSIE), which derives the criterion from the Mean Squared Prediction Error^20^, with its specific computation process delineated as follows:

$$\:MSIE=\frac{1}{n}{\sum\:}_{i=1}^{n}{\left({y}_{i}-{\widehat{y}}_{i}\right)}^{2}$$

where $\:n\:$ is the sample size, $\:{y}_{i}\:$ represents the true (originally observed) blood glucose value, and $\:{\widehat{y}}_{i}$ denotes the imputed value. Lower MSIE values indicate better imputation performance, with zero representing perfect reconstruction of the missing observations. During the application of the adjusted M-H algorithm for imputation, the mean of the incomplete data is designated as the initial value.

The second metric is policy disagreement rate, which quantifies the discrepancy between POMDP strategies implemented with missing data and those executed with complete data by the operational error rate, $\:d\left({\pi\:}_{MDP},\pi\:\right)=\left(\#wactions\right)/\left(\#actions\right)$, where $\:\left(\#wactions\right)$ represents the number of policy disagreement instances (actions) of strategy $\:\:\pi\:\:$ between the imputed-data POMDP and the complete-data POMDP, and $\:\left(\#actions\right)$ denotes the total number of actions evaluated under the complete-data POMDP. A “wrong action” is defined as any instance where the action recommended by the policy trained on imputed data differs from the optimal action recommended by the policy trained on the complete dataset under the same belief state. This metric quantifies policy disagreement between the imputed-data POMDP and the complete-data POMDP under the same pre-specified model structure. It does not represent clinical correctness, insulin safety, or agreement with a clinical ground truth.

The third metric is the absolute reward gap which is calculated as the absolute difference in model-defined cumulative reward between the imputed-data and complete-data trajectories. These three metrics jointly evaluate not only the numerical accuracy of glucose reconstruction, but also the extent to which each imputation method preserves downstream POMDP policy outputs.

### Random missingness scenario

**Fig. 4:**
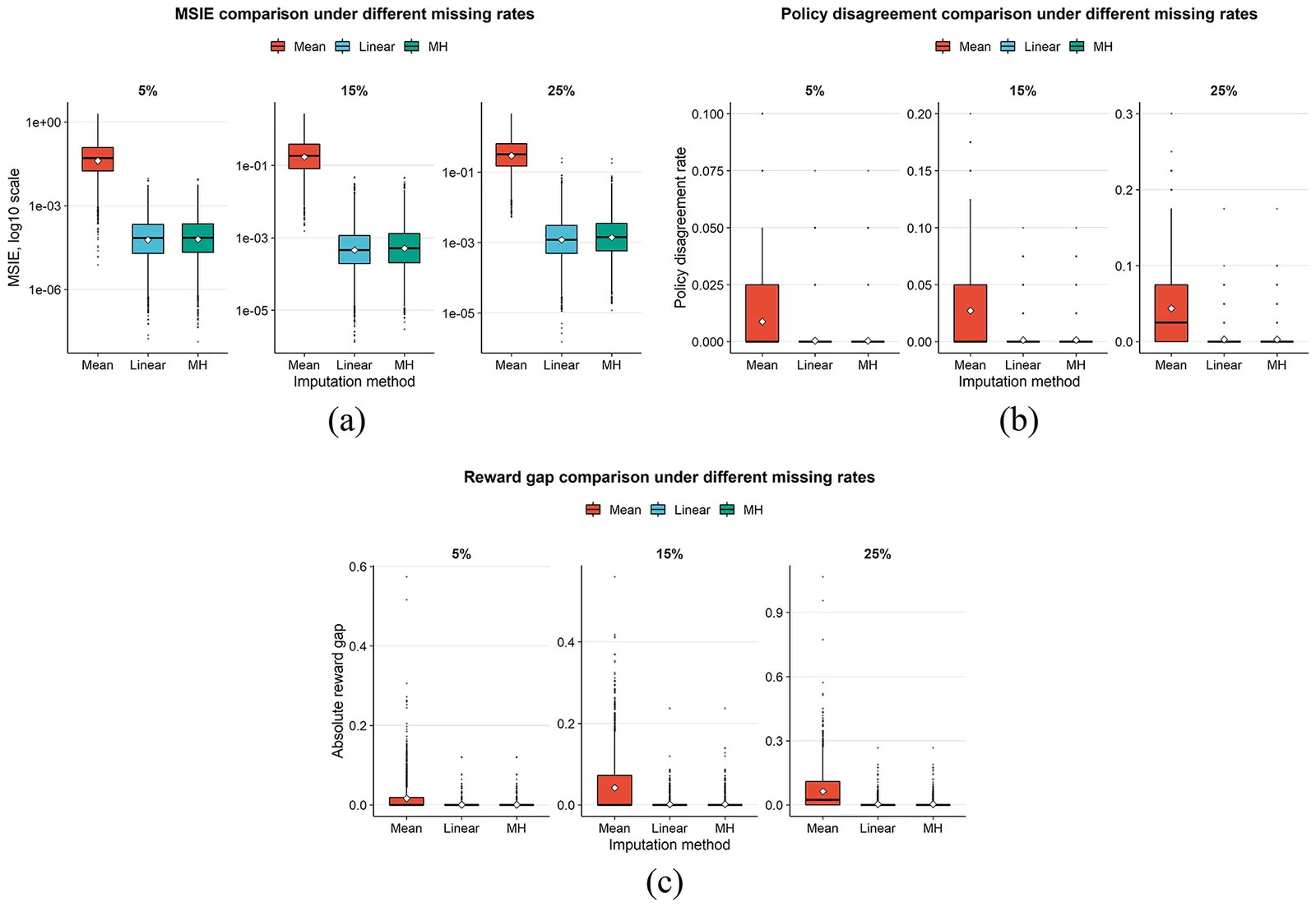
Performance comparison under random missingness. (**a**) MSIE on the log10 scale. (**b**) Policy disagreement rate between the complete-data and imputed-data POMDP policies. (**c**) Absolute reward gap relative to the complete-data benchmark.

We first evaluate the three imputation methods under random missingness, where artificial missing values were randomly generated across each complete CGM sequence at missing rates of 5%, 15%, and 25%. This setting is used to examine the effect of sporadic missing observations on both pointwise glucose reconstruction and downstream POMDP-based decision outputs. Figure 4 summarizes the global comparison of the three methods using MSIE, policy disagreement rate, and absolute reward gap. Because the global boxplots may obscure sequence-specific advantages, we further identified representative CGM sequences in which adjusted M-H achieved lower MSIE than linear interpolation; these locally favorable cases are summarized in Table 4.

**Table 4:** Representative CGM sequences in which adjusted M-H outperforms linear interpolation under random missingness.

Sequence	MH better rate	Linear MSIE	MH MSIE	Improvement
XB48 Quinoa rep1	93.3%	0.001598	0.001233	22.9%
XB48 Rice+Fiber rep2	100.0%	0.001442	0.001142	20.8%
XB95 Berries rep1	80.0%	0.001811	0.001467	19.0%
XB44 Bread + Fat rep1	53.3%	0.002090	0.001794	14.2%
XB70 Pasta+Fiber rep2	80.0%	0.002379	0.002093	12.0%
XB31 Grapes rep1	60.0%	0.005897	0.005457	7.5%

Sequence : a complete CGM trajectory defined by subject ID, food condition, and replicate number in the Stanford CGM dataset; MH better rate: the percentage of paired random-missing trials in which adjusted M-H produced a lower MSIE than linear interpolation for the same sequence; Linear MSIE and MH MSIE: the average MSIE values of the two methods; Improvement: the relative MSIE reduction achieved by adjusted M-H compared with linear interpolation, calculated as $\:(\mathrm{Linear\:MSIE}-\mathrm{MH\:MSIE})/\mathrm{Linear\:MSIE}\times\:100\mathrm{\%}$.

As shown in Fig. 4, mean imputation produces substantially larger reconstruction errors and downstream POMDP deviations than the other two methods. Linear interpolation achieves the lowest global MSIE, while adjusted M-H showed comparable MSIE values and nearly identical POMDP-level outcomes. Specifically, the policy disagreement rates and absolute reward gaps of adjusted M-H are very close to those of linear interpolation across all missing-rate scenarios, indicating that both temporally informed methods preserved the POMDP-derived policy outputs much better than mean imputation.

Although linear interpolation shows a slight advantage in global MSIE, this overall comparison does not fully reflect the situations in which adjusted M-H can provide additional value. Pairwise sequence-level analyses show that adjusted M-H achieves lower MSIE than linear interpolation in a subset of CGM trajectories (see Table 4), especially those with locally nonlinear postprandial patterns. For example, adjusted M-H outperforms linear interpolation in 93.3% of paired trials for the XB48 Quinoa rep1 sequence, reducing the average MSIE from 0.001598 to 0.001233, corresponding to a 22.9% improvement. The advantage of adjusted M-H is also observed in specific high-error trials. Under 25% missingness, adjusted M-H reduces MSIE compared with linear interpolation in several postprandial trajectories, such as XB48 Potatoes rep2, XB31 Grapes rep1, XB19 Potatoes rep3, and XB70 Rice rep1. These trajectories are more likely to contain rapid glucose excursions, delayed peaks, or curved postprandial responses. In such cases, linear interpolation may oversimplify the missing segment by connecting neighboring observations with a straight line. In contrast, adjusted M-H combines a local temporal bridge, a Markovian transition prior, and a smoothness constraint, allowing the imputed values to better follow locally nonlinear glucose dynamics.

From a glucose management perspective, these locally nonlinear periods are clinically and operationally important because they often correspond to postprandial glucose excursions, delayed absorption after mixed meals, or rapid changes in glucose state. Missing values occurring in these periods may have a greater influence on downstream decision support than missing values in stable glucose regions. Therefore, although adjusted M-H does not globally outperform linear interpolation, its local advantage suggests that a model-compatible imputation method may be particularly useful when missing CGM values occur during nonlinear glucose transitions, where preserving state evolution is more important than simple pointwise interpolation.

### Block missingness scenario

**Fig. 5:**
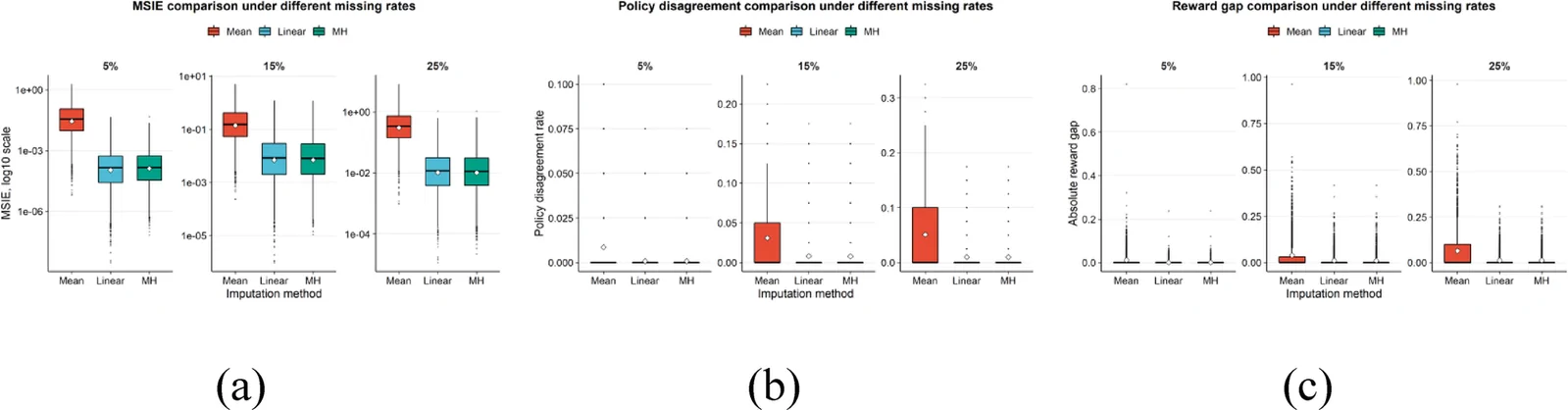
Performance comparison under block missingness. (**a**) MSIE on the log10 scale. (**b**) Policy disagreement rate between the complete-data and imputed-data POMDP policies. (**c**) Absolute reward gap relative to the complete-data benchmark.

In the block missingness scenario, consecutive missing segments are generated to evaluate the robustness of the three imputation methods under short periods of continuous CGM signal loss. The block length is set to 2–6 consecutive observations, while the overall missing rates are maintained at 5%, 15%, and 25%. Figure 5 summarizes the global comparison of mean imputation, linear interpolation, and adjusted M-H in terms of MSIE, policy disagreement rate, and absolute reward gap. We also examine sequence-specific advantages under block missingness by identifying representative CGM trajectories in which adjusted M-H achieved lower MSIE than linear interpolation; these local advantage cases are summarized in Table 5.

**Table 5:** Representative CGM sequences in which adjusted M-H outperforms linear interpolation under block missingness.

Sequence	MH better rate	Linear MSIE	MH MSIE	Improvement
XB94 Beans rep2	66.7%	0.001068	0.000627	41.3%
XB14 Beans rep2	73.3%	0.000803	0.000501	37.6%
XB95 Berries rep2	66.7%	0.001360	0.000927	31.9%
XB91 Potatoes rep1	80.0%	0.002090	0.000143	31.9%
XB70 Pasta + Fat rep2	80.0%	0.000883	0.000620	29.8%
XB68 Rice+Fiber rep2	66.7%	0.003893	0.002938	24.5%

Sequence : a complete CGM trajectory defined by subject ID, food condition, and replicate number in the Stanford CGM dataset; MH better rate: the percentage of paired random-missing trials in which adjusted M-H produced a lower MSIE than linear interpolation for the same sequence; Linear MSIE and MH MSIE: the average MSIE values of the two methods; Improvement: the relative MSIE reduction achieved by adjusted M-H compared with linear interpolation, calculated as $\:(\mathrm{Linear\:MSIE}-\mathrm{MH\:MSIE})/\mathrm{Linear\:MSIE}\times\:100\mathrm{\%}$.

From Fig. 5, we can see that mean imputation again produces the largest reconstruction errors and downstream POMDP deviations. In contrast, linear interpolation and adjusted M-H performed substantially better across all three metrics. Compared with the random missingness scenario, the relative advantage of adjusted M-H becomes more evident under block missingness. At 15% and 25% missingness, adjusted M-H achieved slightly lower global MSIE than linear interpolation, with mean MSIE values of 0.028978 and 0.027706 for adjusted M-H compared with 0.030091 and 0.028403 for linear interpolation, respectively. The two methods also show very similar policy disagreement rates and absolute reward gaps, with adjusted M-H being slightly lower at the higher missing rates.

This pattern is consistent with the structure of the block missingness scenario. When missing observations occur as consecutive segments, linear interpolation reconstructs the entire missing block by drawing a straight line between the two boundary observations. This strategy is effective for short and approximately linear glucose changes, but it may be less accurate when the missing block covers a curved postprandial response, a delayed peak, or a rapid transition between glucose states. By contrast, adjusted M-H uses a local temporal bridge together with a Markovian transition prior and a smoothness constraint. This allows the imputed trajectory to incorporate local nonlinear glucose dynamics and state-transition information rather than relying only on a deterministic straight-line reconstruction.

Pairwise analyses further support this interpretation (see Table 5). adjusted M-H achieves lower MSIE than linear interpolation in 60.0% and 57.4% of paired trials under 15% and 25% block missingness, respectively. Representative sequence-level cases in which adjusted M-H shows local advantages are summarized in Table 5. These cases include meal-response trajectories such as Beans, Bread, Potatoes, Pasta + Fat, and Rice+Fiber, where glucose responses may involve delayed absorption, nonlinear excursions, or gradual postprandial changes. From a glucose management perspective, these consecutive missing segments are important because they may obscure the temporal evolution of glucose states during periods when treatment decisions are more sensitive to trend information. Therefore, the local advantage of adjusted M-H under block missingness suggests that model-compatible imputation may be particularly useful when CGM signal loss occurs during nonlinear or transitional glucose dynamics.

## Conclusions

This study evaluates the impact of missing CGM data imputation on downstream POMDP-based policy outputs using real CGM trajectories from the Stanford Continuous Glucose Monitoring Database. We compare three imputation methods, including mean imputation, linear interpolation, and a bridge-based adjusted M-H algorithm, under two missingness scenarios: random missingness and block missingness. The evaluation is conducted at missing rates of 5%, 15%, and 25% using three metrics: imputation accuracy measured by MSIE, policy disagreement rate, and model-defined reward gap relative to the complete-data benchmark.

The results shows that mean imputation consistently produced the largest reconstruction errors and the greatest deviations in downstream POMDP outputs. In contrast, linear interpolation and adjusted M-H substantially reduce MSIE and better preserved POMDP-derived policy consistency and reward estimates. Under random missingness, linear interpolation achieves slightly lower global MSIE than adjusted M-H, whereas both methods produce nearly identical policy disagreement rates and reward gaps. Under block missingness, adjusted M-H shows more favorable performance, particularly at higher missing rates and in representative CGM trajectories with locally nonlinear postprandial glucose responses. These findings suggest that linear interpolation is a strong baseline for short and smooth missing CGM gaps, while adjusted M-H provides a model-compatible alternative that may be particularly useful when missing values occur during nonlinear glucose transitions or consecutive signal-loss periods.

This study has several limitations. First, although the Stanford CGM data are real measurements, they were collected under standardized food challenge conditions and contain relatively short postprandial trajectories. Therefore, future studies should validate the findings using longer free-living CGM records with more diverse behavioral, dietary, and treatment patterns. Second, the missingness mechanisms are artificially generated to support controlled comparison across imputation methods. Real-world CGM missingness may be informative and related to device use, patient activity, sensor failures, or glucose dynamics. Multiple imputation (MI)^23,24^ is widely used for missing data, particularly under missing-at-random assumptions. In this study, MI was not included because our focus was on single-trajectory imputation methods within a fixed POMDP evaluation framework. Incorporating MI would require additional procedures for aggregating belief updates, policies, and rewards across multiple imputed CGM trajectories, which we leave for future work. Third, the POMDP transition, observation, and reward structures are predefined for scenario-based evaluation. Therefore, the resulting policy outputs should be interpreted as model-defined insulin-intensity categories rather than clinically validated insulin dosing recommendations.

Despite these limitations, this study demonstrates that the choice of imputation method can materially affect both reconstructed CGM trajectories and downstream POMDP decision outputs. The findings indicate that temporally informed imputation methods are preferable to mean imputation when applying POMDP-based models to incomplete CGM data. More importantly, the adjusted M-H algorithm offers a flexible framework for incorporating local temporal structure, Markovian state-transition information, and smoothness constraints into missing-data imputation. This model-compatible structure may be valuable for future sequential decision-support applications in which preserving the temporal evolution of physiological states is essential under incomplete observations.

## Data Availability

The datasets generated and analyzed during the current study are available from the corresponding author upon reasonable request. The data were generated using the AIDA diabetes simulator, an open-access educational tool. Detailed descriptions of the simulation parameters and patient profiles are provided in the Methods section.

## Code availability

The R code used for data imputation (adjusted Metropolis-Hastings algorithm) and for solving the POMDP model (using the POMDP package) is available from the corresponding author upon reasonable request.
